# CCR1-mediated accumulation of myeloid cells in the liver microenvironment promoting mouse colon cancer metastasis

**DOI:** 10.1007/s10585-014-9684-z

**Published:** 2014-10-18

**Authors:** Hideyo Hirai, Teruaki Fujishita, Kazuki Kurimoto, Hitoshi Miyachi, Satsuki Kitano, Susumu Inamoto, Yoshiro Itatani, Mitinori Saitou, Taira Maekawa, M. Mark Taketo

**Affiliations:** 1 Department of Transfusion Medicine and Cell Therapy, Graduate School of Medicine, Kyoto University, Kyoto, Japan; 2 Pharmacology, Graduate School of Medicine, Kyoto University, Kyoto, 606-8501 Japan; 3 Anatomy and Cell Biology, Graduate School of Medicine, Kyoto University, Kyoto, Japan; 4 Reproductive Engineering Team, Institute for Virus Research, Kyoto University, Kyoto, Japan; 5 Department of Surgery, Graduate School of Medicine, Kyoto University, Kyoto, Japan; 6 JST, ERATO, Yoshida-Konoé-Cho, Kyoto, Japan; 7 Center for iPS Cell Research and Application, Kyoto University, Kyoto, Japan; 8 Institute for Integrated Cell-Material Sciences, Kyoto University, Kyoto, Japan; 9 Present Address: Division of Molecular Pathology, Aichi Cancer Center Research Institute, Nagoya, 464-8681 Japan; 10 Present Address: Moores Cancer Center, UCSD, 3855 Health Sciences Drive, San Diego, CA 92093 USA

**Keywords:** Colon cancer, Liver metastasis, CCR1, Neutrophils, Fibrocytes, MMP9, MMP2

## Abstract

To understand colon cancer metastasis, we earlier analyzed a mouse model that developed liver metastasis of cancer cells disseminated from the spleen. We suggested that CCR1^+^ bone marrow (BM)-derived cells are recruited to the microenvironment of disseminated colon cancer cells, and produce metalloproteinases MMP9 and MMP2, helping metastatic colonization. In the present study, we have examined these myeloid cells expressing CCR1 and/or MMPs in detail. To this end, we have established bacterial artificial chromosome (BAC)-based transgenic mouse lines in which membrane-targeted Venus fluorescent protein (mVenus) was expressed under the control of *Ccr1* gene promoter. Then, myeloid cells obtained from the BM and liver metastatic foci were analyzed by the combination of flow cytometry and cytology/immunohistochemistry, in situ RNA hybridization, or quantitative RT-PCR. We have found four distinct types of myeloid cells recruited to the metastatic foci; neutrophils, eosinophils, monocytes and fibrocytes. These cell types exhibited distinct expression patterns for CCR1, MMP2 and MMP9. Namely, neutrophils found in the early phase of cancer cell dissemination expressed CCR1 exclusively and MMP9 preferentially, whereas fibrocytes accumulated in later phase expressed MMP2 exclusively. Either genetic inactivation of *Ccr1* or antibody-mediated neutrophil depletion reduced subsequent recruitment of fibrocytes. The recruitment of CCR1^+^ neutrophils in early phase of colon cancer dissemination appears to cause that of fibrocytes in late phase. These results implicate the key role of CCR1 in colon cancer metastasis in this mouse model, and explain why both MMP9 and MMP2 are essential as genetically demonstrated previously. The results also suggest relevant mechanisms in humans.

**Electronic supplementary material:**

The online version of this article (doi:10.1007/s10585-014-9684-z) contains supplementary material, which is available to authorized users.

## Introduction

The major cause of death for colorectal cancer (CRC) patients is its metastasis to the vital organs such as liver or lungs, like in many other types of cancer. While metastasis formation can be described in about seven successive steps [1], two critical components are invasion and metastatic colonization. Both these processes are carried out through cooperation between the cancer cells and stromal microenvironment [2]. Accumulating evidence indicates that bone marrow (BM)-derived (*i.e.*, myeloid) cells constitute the major components of cancer microenvironment, and play significant roles in the pathophysiology of cancer [3–6]. Namely, tumor-associated macrophages (TAM) and neutrophils (TAN), and myeloid-derived suppressor cells (MDSC) can enhance cancer progression by facilitating the growth and migration of tumor cells, angiogenesis and/or repression of anti-tumor immune responses [7–10]. There is a strong association between the increased density of macrophages or neutrophils in cancer tissues and poor patient survival [11–14]. These results suggest that involvement of myeloid cells is clinically important, and that they may be therapeutic targets against tumor progression.

In a genetically engineered mouse model of invasive colorectal cancer, *cis*-*Apc* ^+/Δ716^ *Smad4* ^+/−^(*Apc/Smad4*) mutant mice, we previously found that BM-derived and CD11b^+^ immature myeloid cells (iMCs) accumulated at the invasion front of the intestinal cancer [15]. We demonstrated that chemokine receptor CCR1 expressed on the iMCs was responsible for their accumulation because one of its specific ligands CCL9 (corresponding to CCL15 in humans) was secreted from the cancer cells. The CCR1^+^ myeloid cells appeared to enhance tumor invasion by producing metalloproteinases MMP9 and MMP2. To investigate the role of the myeloid cells in metastasis, we then employed a liver metastasis model by injection of CCL9-expressing mouse colon cancer cells into the spleen of syngeneic hosts, allowing their dissemination to the liver [16]. Metastatic foci were surrounded by CD11b^+^ myeloid cells that expressed CCR1 and promoted metastasis by producing MMP9 and MMP2. Critical involvement of CCR1, MMP9 and MMP2 was verified experimentally using gene knockout mice lacking these proteins, which suggested possible therapeutic intervention of cancer progression by targeting these molecules. However, the cell lineage and characteristics of these myeloid cells have not been characterized precisely because of the difficulty in their isolation. The aim of this study is to isolate and characterize the tumor-associated myeloid cells that express CCR1 and MMPs. Here we present the results of such an investigation where we have employed CCR1 reporter transgenic mouse technology and CRC liver dissemination models, demonstrating a spatiotemporal orchestration among distinct myeloid cell populations at the metastatic cancer foci.

## Materials and methods

### Mice

As hosts of tumor injections, C57BL/6 wild type or C57BL/6 *Ccr1* ^−/−^ mice [17] were used at 7 week of age. The bacterial artificial chromosome (BAC)-based transgenic mice were generated as described previously [18]. In brief, BACs bearing the *Ccr1* genomic locus of the C57BL/6 mouse strain (WI1-233F4) were purchased from BACPAC Resources Center (Children’s Hospital Oakland Research Institute, Oakland, CA, USA). The gene encoding Venus fluorescent protein targeted to the plasma membrane (mVenus) was recombined immediately after the first in-frame ATG of the *Ccr1* gene exon 2, followed by a polyadenylation sequence using Red/ET Recombineering (Gene Bridges, Heidelberg, Germany), according to the manufacturer’s protocol. We confirmed that no CCR1 protein was produced from the construct. The entire genomic sequence (~42 kb) was excised by Fsp I and purified using Wizard DNA Clean-Up System (Promega). The transgenic founders were established in the C57BL/6 background. All animals were bred and maintained according to the protocol approved by the Animal Care and Use Committee of Kyoto University.

### Experimental metastasis model

Mouse colon cancer cell line CMT93 (of the C57BL/6) was cultured at 37 °C in DMEM with 10 % fetal calf serum (FCS) under 5 % CO_2_. To model liver metastases, 1.5 × 10^6^ of CMT93 cells were injected into the spleen of each C57BL/6 wild type or *Ccr1* ^−/−^ mouse under anesthesia [16]. The spleen was removed 1 min after tumor cell injection. To analyze hematopoietic cells in the metastatic foci, liver tissues were minced, mashed through a cell strainer (pore size; 40 μm) and treated with 0.5 U/ml Liberase DL (Roche Applied Science, Mannheim, Germany) and 5 μg/ml DNase I for 10 min.

### Flow cytometric analysis

Flow cytometric analysis and cell sorting were performed with a FACSCalibur, FACSCanto II, or FACSAria instruments (BD Biosciences, San Jose, CA, USA) as previously described [19]. Cells were stained with allophycocyanin (APC)-conjugated anti-CD11b, phycoerythrin (PE)-conjugated anti-CD45 and PE-Cy7–conjugated anti-Gr-1 antibodies (eBioscience). Cells were stained with propidium iodide (PI) to eliminate dead cells. Data were analyzed with the FlowJo software (Tree Star, Ashland, OR, USA).

### RT-PCR and real-time RT-PCR

Total RNA was extracted with an RNeasy Micro Kit (Qiagen, Valencia, CA, USA) and converted to cDNA using random primers. The cDNA was subjected to real-time PCR using an Applied Biosystems Step One Plus thermal cycler with the following setting: 95 °C for 20 s, followed by 45 cycles of 95 °C for 1 s and 60 °C for 20 s. The sequences for the primers and probes are shown in Supplementary Table 1. The probes were obtained from the Universal Probe Library (Roche Applied Science, Mannheim, Germany). The results were normalized to the levels of *Gapdh* mRNA.

### Histological analyses

The methods for immunohistochemistry were described previously [15]. For immunofluorescence staining, tissues were directly embedded in O.C.T. Compound (Sakura Finetek), and sectioned at 6 μm. The sections were immunostained using the following primary antibodies: Rabbit antibody for rat collagen 1 (L.S.L., Tokyo, Japan); rat monoclonal antibodies for mouse CD34 (RAM34, MEC14.7 and 3H1240), CD45 (BD Biosciences), CD11b (eBiosciences) or Gr-1 (eBiosciences). Antibodies for IgG labeled with Alexa Fluor 488 or Alexa Fluor 594 (Molecular Probes) were used as secondary antibodies. Nuclei were stained with DAPI (Molecular Probes).

### In situ hybridization

We employed the methods published earlier [16–20]. Namely, *Mmp2* cDNA from CMT93 liver metastatic foci was cloned into pSPT18 vector (Roche). Digoxigenin-labeled sense and antisense RNA probes were synthesized with SP6 and T7 RNA polymerase respectively (Roche) and purified with NucAway Spin Columns (Ambion). Sections were cut at 8 µm thickness and hybridized with synthesized probes. DIG-labeled RNA probes were detected by anti–digoxigenin AP Fab fragments (Roche) with NBT/BCIP (Roche).

### Wright Giemsa staining

Smears or cytospin specimens of mouse blood cell samples were stained by a modified Wright Giemsa staining method, using Diff-Quik kit (Sysmex, Kobe, Japan).

### Patients

Clinical samples of metastatic CRC in the liver were obtained from patients who underwent partial liver resection operations at Kyoto University Hospital between January 2006 and December 2010. Colorectal cancer liver metastases were confirmed by pathological examinations. This study protocol was approved by the institutional review board (Ethics Committee) of Kyoto University, Kyoto, Japan, and patients signed the consent forms for the sample use and data analysis.

### Statistics

Statistical significance was evaluated with the Student’s *t* test. The *P* values <0.05 were considered as statistically significant. Each data set is represented as the mean ± SD.

## Results

### Generation of *Ccr1*-mVenus transgenic mice and analysis of BM cells

**Fig. 1:**
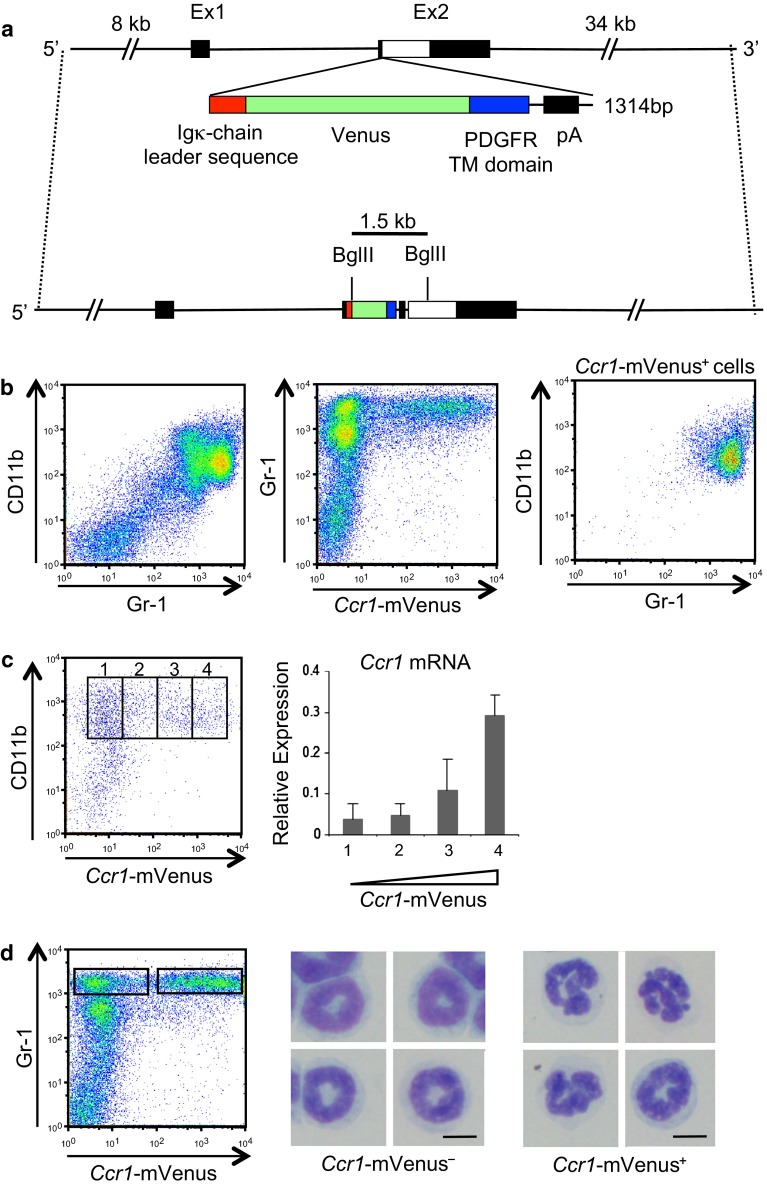
Expression of *Ccr1*-mVenus reporter is restricted to Gr-1^+^ mouse neutrophils. **a** Schematic illustration of the bacterial artificial chromosome (BAC) carrying mouse *Ccr1* gene. A gene encoding Venus targeted to plasma membrane (mVenus) was recombined in frame with the first ATG in exon 2 of *Ccr1* followed by a polyadenylation sequences, so that no functional CCR1 is expressed from the BAC transgene. It carried ~8 kb of upstream and ~34 kb downstream genomic sequences from the *Ccr1* gene locus. PDGFR TM domain: platelet-derived growth-factor receptor transmembrane domain. pA: polyadenylation signal. Ex: exon. **b** Expression of *Ccr1*-mVenus reporter gene specifically in the Gr-1^+^ neutrophils. Presented are flow cytometric profiles of BM cells from one of the four *Ccr1*-mVenus reporter transgenic lines (line #15; see also Supplementary Fig. 1). Most Gr-1^+^ cells are CD11b^+^ (*left*) and a subpopulation of Gr-1^+^ cells express *Ccr1*-mVenus (*center*). In the *right panel*, cells within the *Ccr1*-mVenus^+^ gate are shown. **c** Expression of *Ccr1*-mVenus reporter correlated well with the abundance of endogenous CCR1 transcripts. CD11b^+^ cells were divided into four populations based on the expression level of *Ccr1*-mVenus reporter (1–4 in the *left panel*), and RNA extracted from each population was subjected to real-time RT-PCR for quantification of endogenous *Ccr1* transcripts (*right*). Results are shown as the mean ± S.D. (*n* = 3). **d** *Ccr1*-mVenus reporter is expressed by mature Gr-1^+^ neutrophils in the BM. *Ccr1*-mVenus^–^ and CCR1-mVenus^+^ BM cells were sorted (*left*) and subjected to modified Wright Giemsa staining of the cytospin specimens (*center* and *right*). *Scale bars*, 10 μm

We demonstrated recently that CCR1 plays critical roles in liver metastasis of Smad4-deficient colon cancer in a mouse model [16]. In the BM, CD11b^+^ Gr-1^+^ myeloid cells expressed 10 times higher level of *Ccr1* mRNA than CD11b^–^ Gr-1^–^ non-myeloid cells (Supplementary Fig. 1a), suggesting that CCR1 expressing cells were highly enriched in the myeloid cells. To isolate and characterize the CCR1-expressing cells by cell sorting, we tested antibodies from various sources, but were unable to find one that bound to mouse CCR1 specifically and reliably. Accordingly, we resorted to the construction of a reporter transgenic mouse model whose marker gene (membrane-targeted Venus; mVenus) was placed under the control of the *Ccr1* promoter. As the source of regulatory elements to reconstitute the endogenous CCR1 expression, we used a BAC clone spanning 8 kb upstream and 34 kb downstream of the mouse *Ccr1* gene (Fig. 1a). Thus, we established four independent transgenic lines (Fig. 1b, and Supplementary Fig. 1b, c; see "Materials and methods" section).

A flow cytometric analysis of adult transgenic mouse BM cells showed that expression of the reporter mVenus fluorescence was restricted essentially to the CD11b^+^ Gr-1^+^ myeloid cells (Fig. 1b and Supplementary Fig. 1c). When the CD11b^+^ myeloid cells were divided into four populations based on the intensity of mVenus fluorescence, the level of endogenous *Ccr1* mRNA correlated well with that of mVenus fluorescence, suggesting that expression of the reporter precisely reflected that of the endogenous CCR1 (Fig. 1c). Notably, CD11b^+^ Gr-1^+^ myeloid cells could be divided into *Ccr1*-mVenus^–^ and *Ccr1*-mVenus^+^ cells (Fig. 1d, left panel). Giemsa staining of the sorted cells in these subpopulations revealed that the *Ccr1*-mVenus^–^ cells were immature neutrophils with ringed nuclei, whereas the *Ccr1*-mVenus^+^ cells were mature neutrophils with lobulated nuclei, indicating that expression of CCR1 correlated with the maturation of BM neutrophils (Fig. 1d, center and right).

### Accumulation of CCR1^+^ cells in mouse liver metastatic foci

**Fig. 2:**
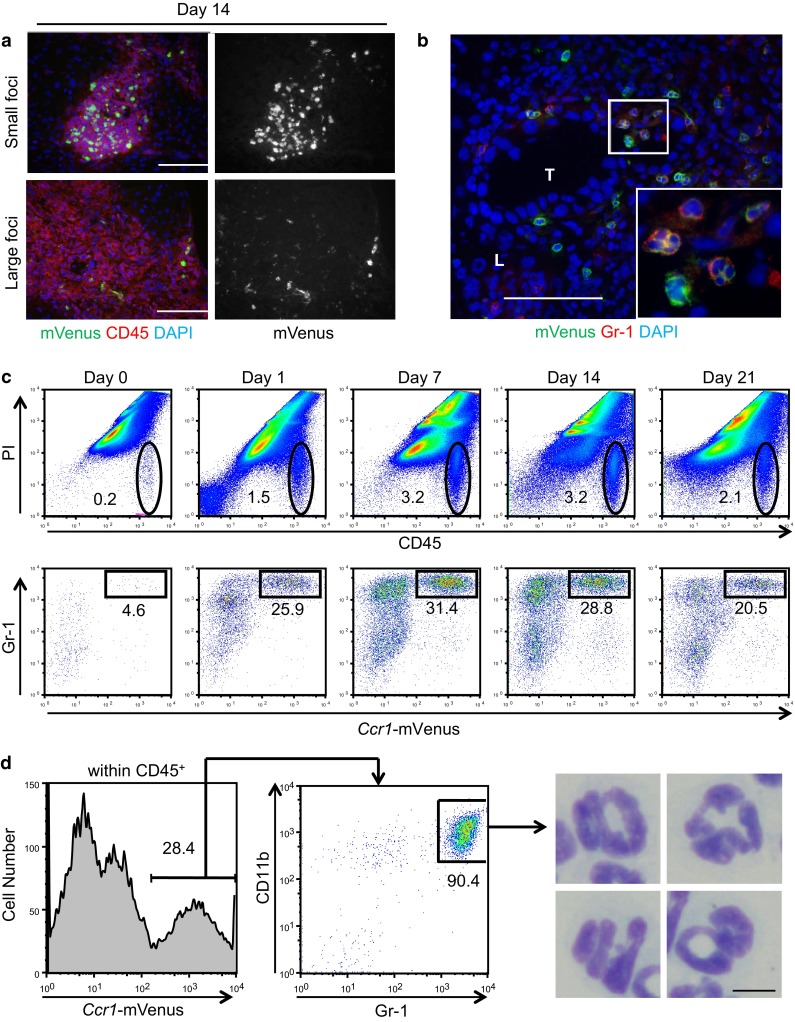
Most CCR1^+^ cells accumulating in the liver metastatic foci are neutrophils.(**a** and **b**) Immunofluorescence analysis of the liver metastatic foci on day 14 post-transplantation. Expression of CD45 (a pan-leukocyte marker) and *Ccr1*-mVenus reporter are shown for small (*upper photos*) and large (*lower photos*) metastatic liver foci (**a**). A large metastatic liver focus analyzed for expression of *Ccr1*-mVenus and Gr-1 (a mouse neutrophil marker) (**b**). Nuclei were stained with DAPI. Scale bar, 100 μm. T: tumor, L: liver tissue. **c** Chronological changes in the proportion of CD45^+^ hematopoietic cells accumulated in the metastatic foci of the liver. Cells harvested from the liver at the indicated days were analyzed by flow cytometry. Propidium iodide (PI)^−^ CD45^+^ live hematopoietic cells circled in the upper panels were further analyzed for expression of *Ccr1*-mVenus and Gr-1 in the lower panels. Cells outside the circles were hepatocytes, resident stromal cells and/or debris, and therefore, excluded from further analyses. Numbers in the panels show the percentages of CD45^+^ cells (*top*) or *Ccr1*-mVenus^+^ cells within the CD45^+^ cells (*bottom*). **d** Most *Ccr1*-mVenus^+^ cells in the liver at day 14 after tumor transplantation were CD11b^+^ Gr-1^+^ (*left* and *center*), and showed the morphology characteristic of mature neutrophils (*right*). *Scale bar*, 10 μm

As we reported previously [16], intrasplenic injection of CCL9-expressing CMT93 colon cancer cells can cause their dissemination into the liver, which is followed by metastatic colonization. Employing this model, we monitored the behavior of the *Ccr1*-mVenus^+^ cells in the cancer metastasis microenvironment first by histochemistry at 14 days post-injection. We found that the density of *Ccr1*-mVenus^+^ cells among CD45^+^ hematopoietic cells was relatively high (~50/0.01 mm^2^) in small foci (i.e., Φ < 0.2 mm; Fig. 2a, top) whereas it was very low (<5/0.01 mm^2^) in large foci (Φ > 0.2 mm; Fig. 2a, bottom). These results suggest that accumulation of CCR1^+^ cells at the metastatic foci takes place at earlier stages around micrometastases rather than at later stages of colonization. At a higher magnification, 50–80 % of the *Ccr1*-mVenus^+^ cells were Gr-1^+^ neutrophils, whereas the remaining Gr-1^−^ cells contained both mononuclear and non-mononuclear cells (Fig. 2b).

To characterize these myeloid cells further, we then dissociated the liver tissue through the course of dissemination-metastasis and analyzed recovered cells by flow cytometry. Because not all myeloid cells of the liver foci could be collected into cell suspensions even after multiple enzymatic digestions, the remaining CD45^+^ hematopoietic cells in the remnants were also analyzed histochemically (Supplementary Fig. 1d; see below). Before injection of the CRC cells into the spleen, we found only ~0.2 % of the recovered liver resident cells to be propidium iodide (PI)^−^ and CD45^+^ live hematopoietic cells, and only ~5 % of them were *Ccr1*-mVenus^+^ with their majority being Gr-1^+^ (Fig. 2c, Day 0). On day 1 post-injection, CD45^+^ hematopoietic cells began to accumulate in the liver and peaked between days 7 and 14, comprising up to ~3 % of recovered cells with their 20–30 % being *Ccr1*-mVenus^+^ (Fig. 2c). These *Ccr1*-mVenus^+^ cells were mostly neutrophils as identified by their expression of CD11b and Gr-1, and lobulated nuclei (Fig. 2d), which was consistent with the above observation of BM cells (Fig. 1d). Namely, CCR1^+^ myeloid cells in the liver foci were mostly neutrophils when traced after *Ccr1*-mVenus expression.

### Myeloid cells accumulated in liver metastatic lesions include ‘Fibrocytes’

**Fig. 3:**
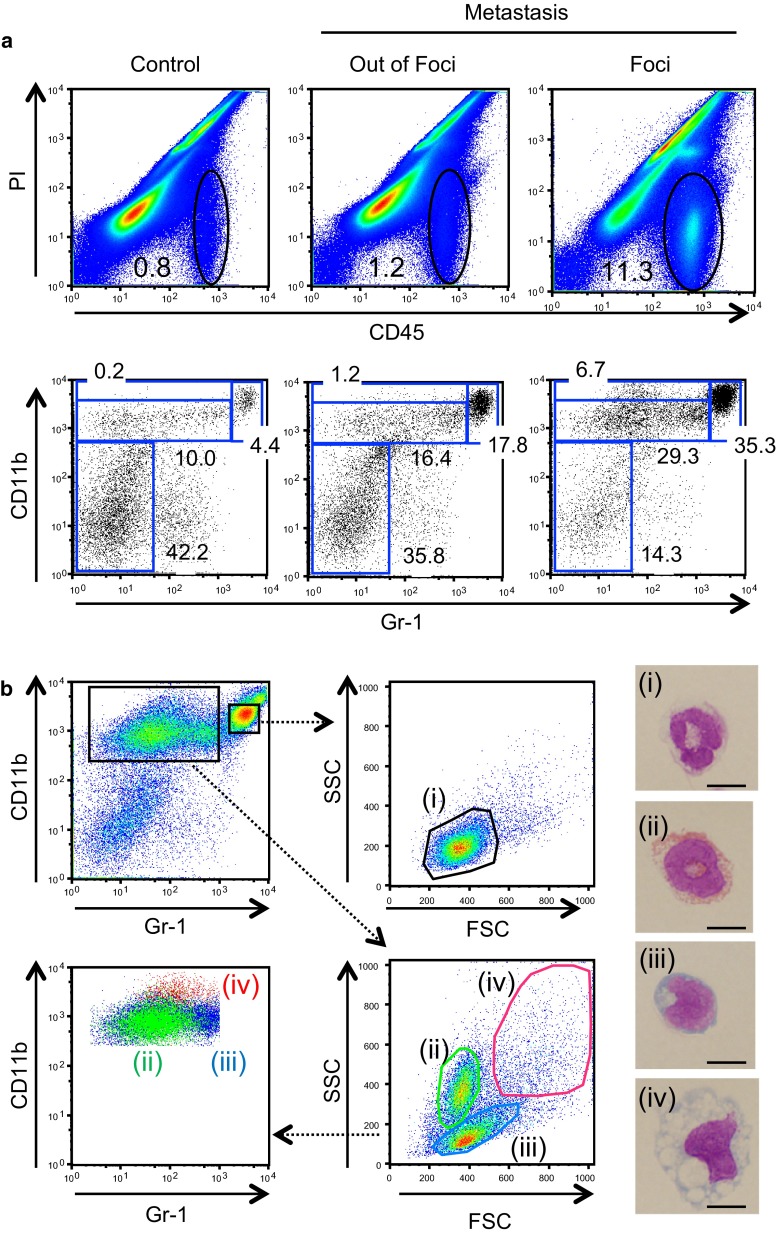
Accumulation of four distinct types of myeloid cells in the metastatic liver foci **a** Cells were isolated from the liver, on or off the foci, on day 14 post-inoculation, and were analyzed by flow cytometry. CD45^+^ hematopoietic cells circled in the upper panels were further analyzed for expression of CD11b and Gr-1 in the lower panels. Numbers in panels are the percentage of CD45^+^ cells (*top*) or those of the gated subpopulations within CD45^+^ cells (*bottom*). **b** Identification of four distinct types of myeloid cells from the metastatic liver foci. The CD45^+^ cells collected from the metastatic foci on day 14 were analyzed for CD11b and Gr-1 (*top left*). CD11b^+^ Gr-1^high^ cells (*top center*) and CD11b^+^ Gr-1^low^ cells (*lower center panel*) were further analyzed for the forward and side scatters (FSC and SSC, respectively). *Lower left panel* shows the distribution of cell populations ‘(ii)–(iv)’ re-analyzed for expression of CD11b versus. Gr-1. Cytospin specimens of four distinct populations (i)–(iv) were subjected to Wright-Giemsa staining (*right*). *Scale bars*, 10 μm

We then analyzed the myeloid cells in the liver metastatic foci in more detail. In large metastatic foci (Fig. 2a), most CD45^+^ hematopoietic cells lacked expression of *Ccr1*-mVenus, although a few expressing cells were also found. To further characterize the hematopoietic cells accumulated at the site of metastasis, we recovered cells from livers of control mice, and from livers with or without metastatic foci, and analyzed them by flow cytometry (Fig. 3a). The live hematopoietic (PI^−^ CD45^+^) fraction was significantly larger in the metastatic foci (~11 vs. ~1 % in non-transplanted control, Fig. 3a top), and contained more CD11b^+^ myeloid cells (~70 vs. ~15 % in the control, Fig. 3a bottom). Then, CD11b^+^ myeloid cells were divided into two subpopulations in terms of Gr-1 expression (Gr-1^high^ and Gr-1^low^; Fig. 3b, top left). The Gr-1^high^ cells clustered as a distinct population in the scatter analysis (FSC vs. SSC; Fig. 3b, top right), and showed the neutrophil morphology in the sorted specimens [Fig. 3b, Photo (i)]. On the other hand, the Gr-1^low^ cells were constituted of three subpopulations in the scatter analysis and re-analysis of the data sets for CD11b vs. Gr-1 (Fig. 3b, bottom). When sorted, two subpopulations with lower scatters (FSC^low^ SSC^low^) showed morphologies of eosinophils and monocytes (Fig. 3b, right photos (ii) and (iii), respectively). In addition, we found cells with a profile of high FSC and SSC with the expression level of CD11b higher than that of eosinophils or monocytes (Fig. 3b, bottom left). The Giemsa staining of the sorted specimens revealed large cells with expanded cytoplasm containing vacuoles, suggesting that they are of the monocyte-macrophage lineage (Fig. 3b, right photo (iv)). We found that all these four subsets of CD11b^+^ myeloid cells were of the BM origin because liver metastasis experiments following BM transplantation using congenic donor cells (CD45.1 as the marker [21]) proved their donor origin (Supplementary Fig. 2).

**Fig. 4:**
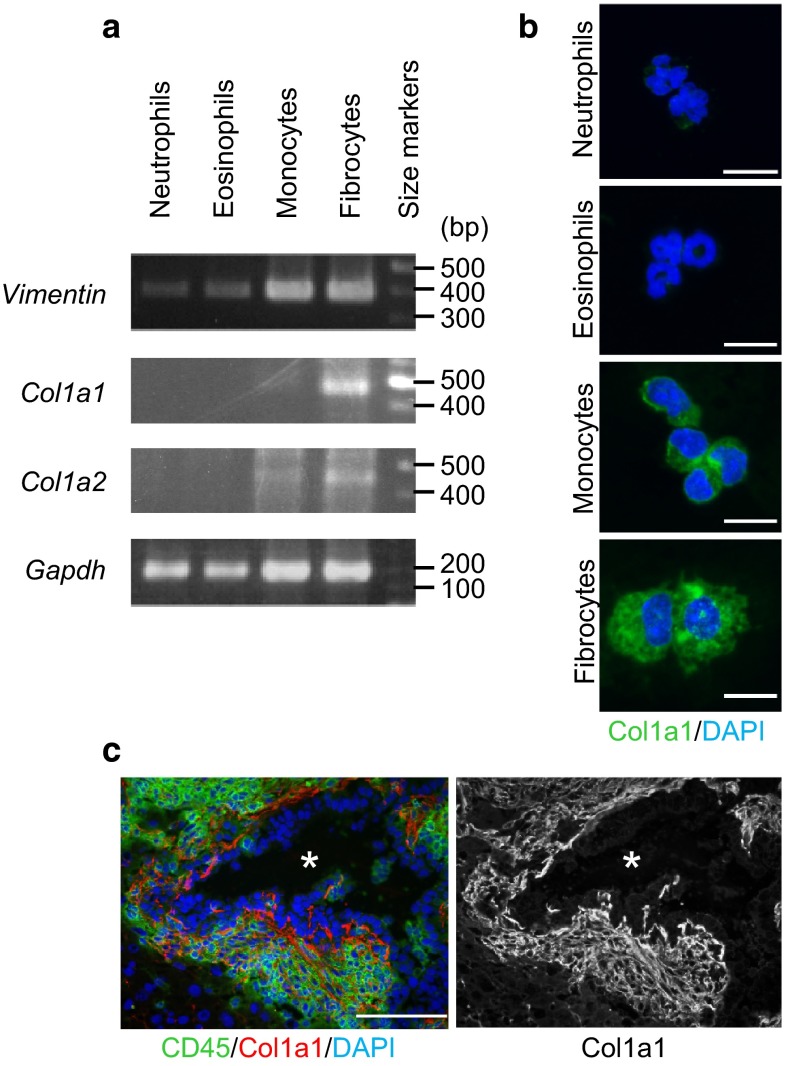
Fibrocyte characteristics exhibited by the macrophage-like cells recovered from the liver metastatic foci (**a**) Quantification of mRNAs for vimentin and collagens by RT-PCR. Myeloid cell subpopulations; neutrophils, eosinophils, monocytes and fibrocytes were collected by cell sorting, and analyzed by RT-PCR. *Gapdh* mRNA is shown as the loading control. **b** Photomicrographs of the sorted subpopulations that were immunostained for collagen, type I α1 (Col1a1). Nuclei were stained with DAPI. *Scale bars*, 10 μm. **c** Immunohistochemical staining of a metastatic liver focus for collagen, type I α1 (Col1a1; *right*) together with CD45 and DAPI (*left*). Note the strong expression of collagen, type I that formed similar structures to the ‘cap’ formation surrounding the metastatic foci [15]. *Scale bar*, 100 μm. Col1a1: collagen type I α1, Col1a2: collagen type I α2

It is known that a monocyte-derived lineage identified as ‘fibrocytes’ expresses a series of mesenchymal molecules as vimentin and collagens together with markers of hematopoietic cells [22]. We found that these monocytes and macrophage-like cells obtained from the metastatic foci expressed such non-hematopoietic marker molecules at substantial levels whereas neutrophils or eosinophils did not (Fig. 4a). Namely, immunofluorescence microscopy of the sorted cells confirmed expression of collagen type I by monocytes and macrophage-like cells derived from the metastatic foci (Fig. 4b). Moreover, most CD45^+^ hematopoietic cells that formed the ‘cap’ [15, 16] around the liver metastatic foci expressed abundant collagen type I (Fig. 4c). Therefore, we identify these macrophage-like cells as fibrocytes. As we reported earlier, these ‘cap’ cells were stained with a particular anti-CD34 antibody (clone: RAM34, Supplementary Fig. 3). However, we recently found that they were not stained with either of two other anti-CD34 antibodies from different sources (MEC14.7 or 3H1240), suggesting that the staining with RAM34 was caused by a non-specific binding of the antibody, conceivably to collagens. In fact, a qRT-PCR analysis of each sorted myeloid population showed that they contained little CD34 mRNA (data not shown). As mentioned above, it was difficult to dissociate and collect fibrocytes from the liver foci due to abundant collagen fibers in the ECM (Supplementary Fig. 1d). Accordingly, fibrocytes did not constitute the major population when isolated cells were analyzed by flow cytometry (see also below).

Collectively, these results indicate that the CD45^+^ hematopoietic cells accumulated around the metastatic foci consist of neutrophils, eosinophils, monocytes and fibrocytes, and that the major types are monocytes and fibrocytes, with the latter producing abundant collagen.

### Chronology of myeloid cell accumulation in liver metastatic foci

**Fig. 5:**
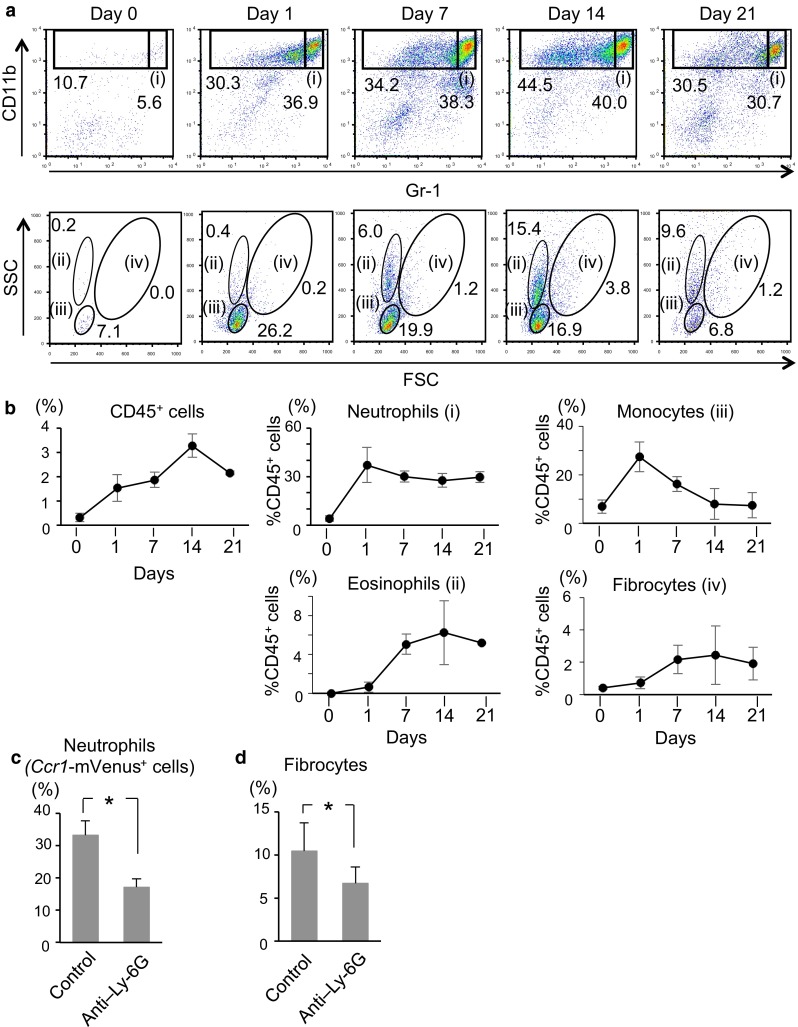
Chronological changes in the myeloid cell distribution in the liver metastatic foci. **a** Flow cytometric analysis of the CD45^+^ cells collected from the metastatic liver foci, performed on days 0–21 post-transplantation for expression of CD11b and Gr-1 (*top*). CD11b^+^ Gr-1^low^ cells were further divided into three distinct populations; eosinophils (ii), monocytes (iii) and fibrocytes (iv) (*bottom*). **b** Results in (**a**) are redrawn as the subpopulation kinetics in the percentage of all CD45^+^ cells. Note that the accumulation of neutrophils and monocytes preceded that of fibrocytes and eosinophils in the metastatic liver foci in this mouse model. Results are shown as the mean ± S.D. (*n* = 3–7) (**c** and **d**) Fibrocyte accumulation was attenuated by neutrophil depletion. An anti–Ly-6G rat monoclonal antibody (1A8) was injected *ip* (500 μg/kg) on days −1 and +3. On day 0, CMT93 syngeneic colon cancer cells were transplanted to C57BL/6 hosts. On day 7, metastatic tissues in the liver were harvested and analyzed by flow cytometry. *Ccr1*-mVenus^+^ cells and fibrocytes among the CD45^+^ cell population were quantified in (**c**) and (**d**), respectively. Results are presented as the mean ± S.D. (*n* = 3). *P < .05 compared with controls

Having identified four distinct myeloid cell types accumulating in the metastatic foci, we investigated the chronological changes in myeloid cells found in the liver metastasis lesions (Fig. 5a, b). The top panels in Fig. 5a show expression of CD11b and Gr-1 among the CD45^+^ hematopoietic cells whereas the bottom panels do FSC and SSC profiles of the CD11b^+^ Gr-1^low^ population. After splenic injection of CMT93 cells, the number of CD45^+^ hematopoietic cells started to increase on day 1 and peaked on day 14, decreasing thereafter. Neutrophils and monocytes rapidly appeared on day 1 at high numbers before the appearance of eosinophils and fibrocytes. Accumulation of eosinophils and fibrocytes began on day 7 and peaked on day 14. These data suggest that the recruitment of neutrophils and monocytes may have caused that of eosinophils and fibrocytes.

To determine the possible role of neutrophils in the emergence of fibrocytes, we performed antibody-mediated neutrophil depletion experiments in vivo. Administration of anti–Ly-6G antibody significantly decreased the number of neutrophils in the BM and *Ccr1*-mVenus^+^ neutrophils in the metastatic foci on Day 7 (Supplementary Figs. 4, 5c). At the same time, this treatment caused a significant decrease in the fibrocyte number as well (on Day 7, Supplementary Fig. 5, 5d), suggesting that the preceding accumulation of neutrophils at around day 1 was necessary for that of fibrocytes later at 7–10 days.

### Roles of CCR1 in myeloid cell accumulation to liver lesions of crc metastasis

**Fig. 6:**
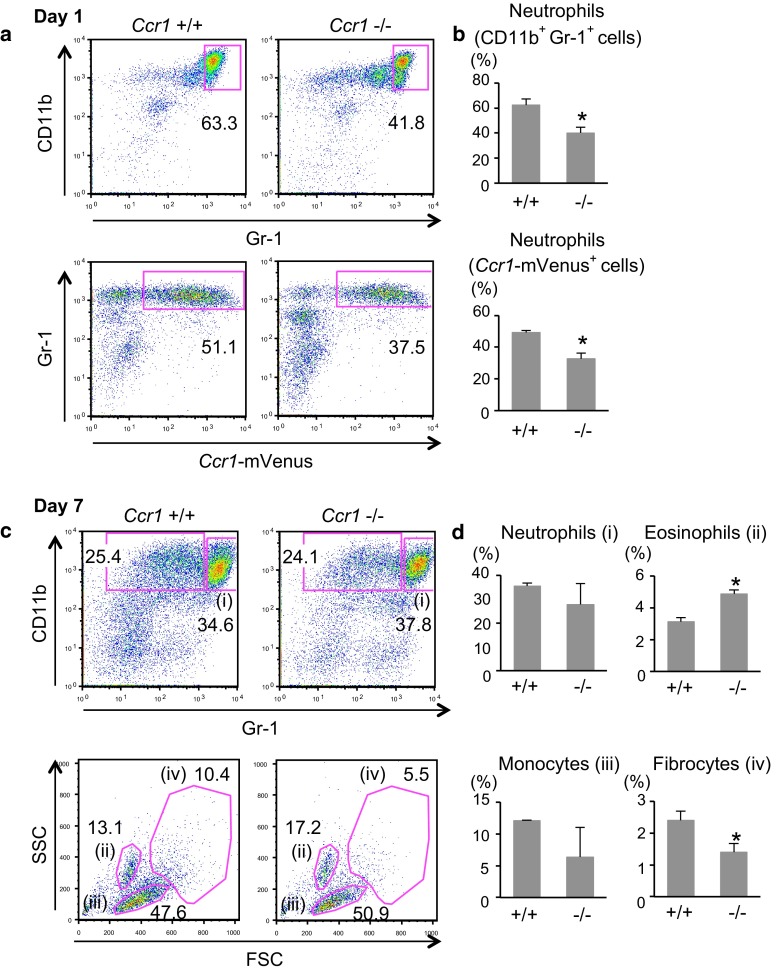
CCR1-dependent accumulation of neutrophils and fibrocytes (**a**) Flow cytometric analysis of the CD45^+^ cells harvested from metastatic liver foci on day 1 post-transplantation of the tumor cells into *Ccr1* ^+/+^ or *Ccr1* ^−/−^ host mice. Numbers indicate the percentages of the gated cells within the whole CD45^+^ cell population. The proportions of CD11b^+^ Gr-1^+^ cells and *Ccr1*-mVenus^+^ cells are summarized in (**b**), (*top* and *bottom*, respectively). Results are presented as the mean ± S.D. (*n* = 3). *P < .05 compared with +/+. **c** Flow cytometric analysis of the CD45^+^ cells harvested from metastatic liver foci on day 7 post-transplantation (*top*). The CD11b^+^ Gr-1^low^ cells were analyzed further for scatter characteristics (FSC vs. SSC; *bottom*). Numbers indicate the percentages of the gated cells. **d** The frequencies for neutrophils (i), eosinophils (ii), monocytes (iii), and fibrocytes (iv) are summarized as the mean ± S.D. (*n* = 4). *P < .05 compared with +/+

To investigate the roles of CCR1 in the accumulation of these myeloid cell types, we introduced the *Ccr1*-mVenus reporter transgene into *Ccr1* ^−/−^ mice by successive crosses and subjected them to liver dissemination-metastasis experiments (Fig. 6). On day 1 post-splenic injection, we observed rapid accumulation of *Ccr1*-mVenus^+^ neutrophils (CD11b^+^ Gr-1^high^) in *Ccr1* ^+/+^ control mice. In the *Ccr1* ^−/−^ mutant mice, however, accumulation of *Ccr1*-mVenus^+^ CD11b^+^ Gr-1^high^ neutrophils was reduced significantly (Fig. 6a, b). On day 7, the relative abundance of neutrophils (*Ccr1*-mVenus^+^ CD11b^+^ Gr-1^high^ cells) between wild type and knockout mice did not differ significantly (*P* = 0.32, Fig. 6c, d), whereas the proportion of eosinophils was increased in *Ccr1* ^−/−^ mice, with that of monocytes and fibrocytes rather decreased (Fig. 6c, d). These results suggest that CCR1 played key roles not only in the earlier mobilization and accumulation of neutrophils, but also in later accumulation of monocytes and fibrocytes in liver lesions of CRC metastasis, although the molecular mechanisms by which neutrophils recruit fibrocytes remain unclear.

### Neutrophils and fibrocytes promote liver metastasis of CRC Cells through expression of MMP9 and MMP2, respectively

**Fig. 7:**
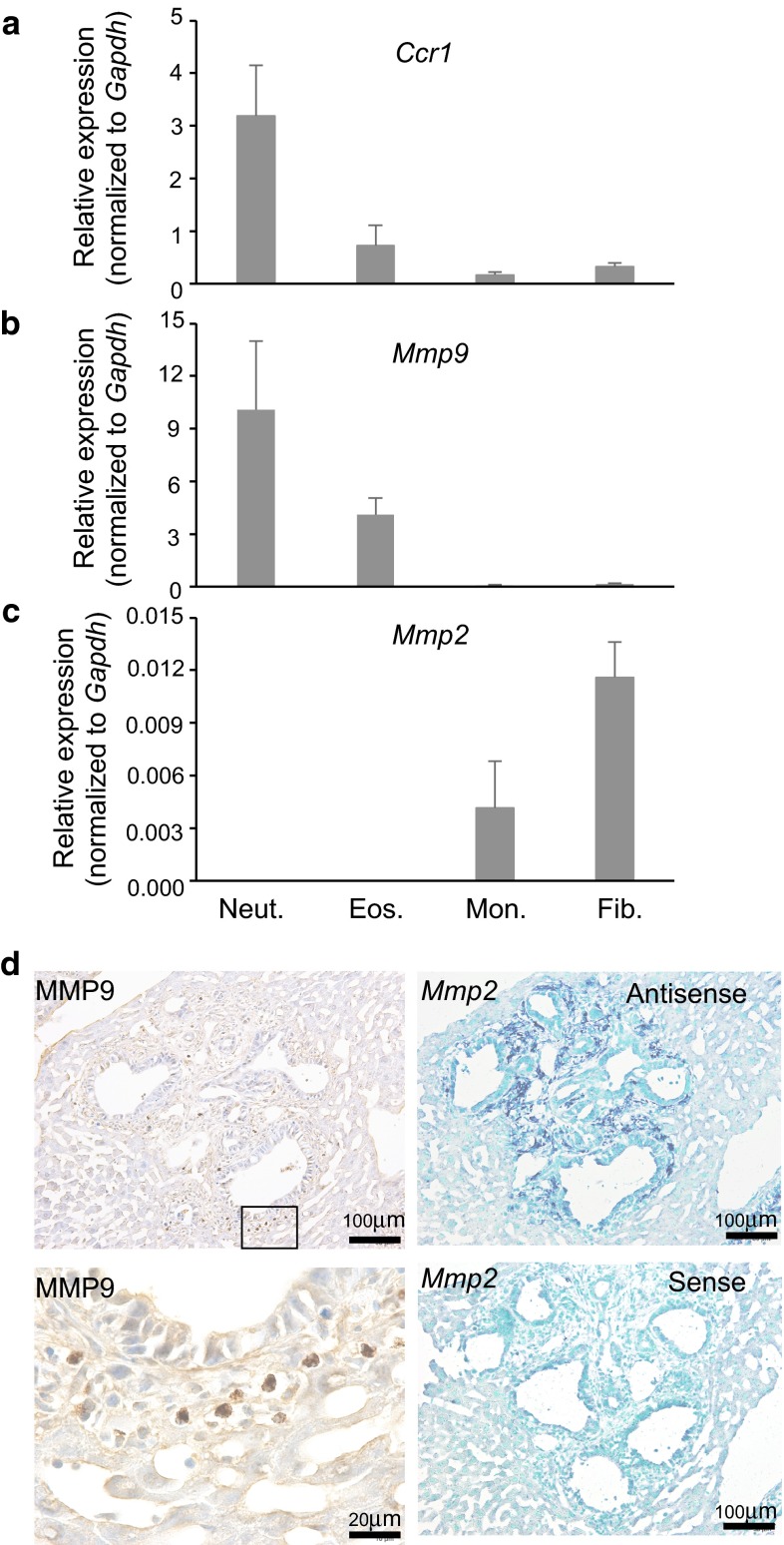
Neutrophils and fibrocytes facilitate liver metastasis by producing metalloproteinases, MMP9 and MMP2, respectively. **a**–**c** Myeloid cell subpopulations isolated from metastatic foci on day 14 post-transplantation were subjected to real-time RT-PCR quantification of mRNAs encoding *Ccr1* (**a**), MMP9 (**b**), or MMP2 (**c**). Results are presented as relative ratios to the level of *Gapdh* mRNA, with the mean ± S.D. (*n* = 3) Neut., neutrophils; Eos., eosinophils; Mon., monocytes; Fib., fibrocytes. **d** Distinct localization of cells expressing MMP9 or MMP2 in the metastatic foci. Serial sections from the liver metastatic foci on day 14 were subjected to immunohistochemistry for MMP9 (*left*) or in situ hybridization for MMP2 mRNA (*right*). *Lower left* photo is a higher magnification of the boxed area in the *upper left*. *Scale bar*, 100 μm

Both MMP9 and MMP2 are required for promotion of liver metastasis of colon cancer in a mouse model [16] To identify the cell type(s) that produce these matrix metalloproteinases, we performed quantitative RT-PCR analyses on the sorted myeloid cells isolated from the liver metastatic foci (Fig. 7a). As anticipated from the results of the *Ccr1*-mVenus reporter transgenic mice, CCR1 was abundantly expressed by neutrophils, with much lower levels in other myeloid cells. On the other hand, metalloproteinases MMP9 and MMP2 were expressed in neutrophils and monocytes/fibrocytes, respectively, at significant levels. Notably, MMP9 and MMP2 were not expressed simultaneously in any of these myeloid cell types (Fig. 7b, c). To further characterize the cell types that express MMP9 or MMP2, we performed immunohistochemistry for MMP9, and in situ hybridization for MMP2 on serial sections of the metastatic mouse livers (Fig. 7d). As the result, MMP9 was expressed only by a limited number of cells with lobulated nuclei (polymorphonuclear cells). In contrast, MMP2 mRNA (that hybridized to the anti-sense oligonucleotide, but not to the sense oligonucleotide) was found in the majority of tumor-associated stromal cells. These results suggest that, in the liver metastatic lesions, MMP9 and MMP2 were produced by neutrophils and monocyte/fibrocytes, respectively; by distinctly different myeloid cell types.

### Human CD15^+^ cells accumulating at liver metastatic lesions express CCR1

In order to seek the clinical relevance of these findings in mice, we investigated expression of CCR1 in human leukocytes. In sharp contrast to mouse neutrophils, flow cytometric analysis revealed that CCR1 was not expressed in human neutrophils, but expressed only by monocytes in the peripheral blood (*n* = 7, Supplementary Fig. 6a). These findings were confirmed by immunofluorescence staining of peripheral blood cytospin specimens. All the cells expressing CCR1 were CD14^+^ monocytes, not CD15^+^ neutrophils (Supplementary Fig. 6b). We further analyzed human peripheral blood for the so-called ‘myeloid-derived suppressor cells’ (MDSC; CD33^+^ HLA-DR^−^ population [23] ), and found that their monocyte (CD14^+^ cells) subpopulation expressed CCR1 (data not shown). In the liver metastatic lesions of human colon cancer, on the other hand, only a small fraction (~10 %) of CD14^+^ monocytes expressed CCR1 by immunohistochemistry, whereas most of the CD15^+^ myeloid cells with mononuclear morphology expressed CCR1 (Supplementary Fig. 6c). These results suggest the possibility that recruitment of MDSC and/or up-regulation of CCR1 in CD15^+^ myeloid cells contribute to liver metastasis of CRCs in humans as well.

## Discussion

**Fig. 8:**
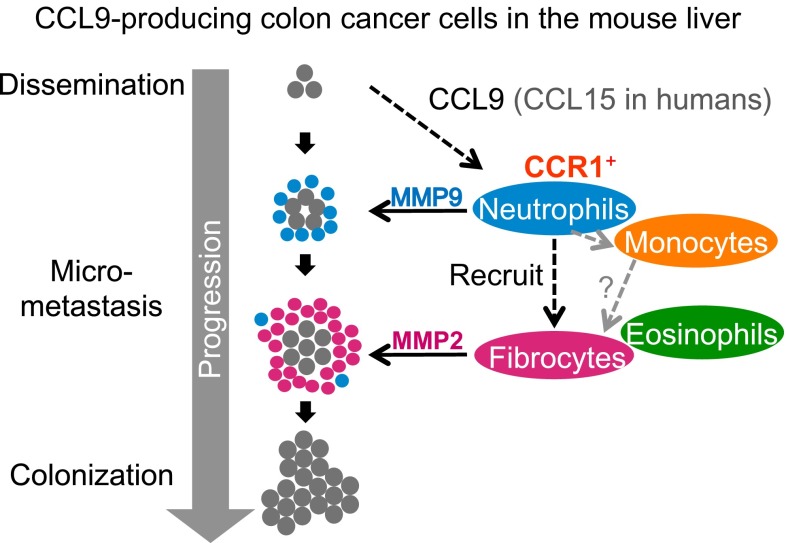
A schematic representation on the roles of myeloid cells in promoting colon cancer liver metastasis. After CCL9-expressing mouse colon cancer cells (CMT93) are allowed to disseminate to the liver, CCR1^+^ host myeloid cells (mostly neutrophils) are recruited to the cancer cell foci in the early phase (~1 day). Neutrophils produce MMP9 and help cancer foci to expand, and at the same time recruit fibrocytes in the late phase (1–2 weeks). Fibrocytes express MMP2 and help further expansion (colonization) of the cancer foci

In the present study, we have demonstrated that nearly all CCR1 expressing cells accumulating at the metastatic foci are neutrophils (Figs. 1, 2). CCR1 was indeed critical for the recruitment of neutrophils, because early (day 1) accumulation of *Ccr1*-mVenus^+^ Gr-1^+^ neutrophils was significantly compromised in *Ccr1* ^−/−^ mutant mice (Fig. 6a). In addition, recruitment of monocyte/fibrocytes to the metastatic foci on day 7 was also reduced in *Ccr1* ^−/−^ mice. Chronologically, accumulation of neutrophils took place earlier than that of fibrocytes (Fig. 5). Moreover, antibody-mediated depletion of neutrophils caused a significant reduction in the fibrocyte number at the foci on day 7 (Supplementary Fig. 5a, b). Collectively, these results suggest that CCR1-mediated early accumulation of neutrophils triggers the fibrocyte recruitment at later stages. It is conceivable that neutrophils either recruited fibrocytes directly or stimulated monocytes to be converted to fibrocytes, or both (Fig. 8).

Because MMP9 and MMP2 act on common substrates, the requirement for both MMPs in iMCs has been an outstanding question. We have now found that CCR1 and MMP9 are expressed preferentially by neutrophils, whereas MMP2 is expressed exclusively by monocytes/fibrocytes. We have also found chronological difference in the recruited BM cell types in liver metastatic foci; early neutrophils and later fibrocytes. These results suggest that production of MMP9 and MMP2 in succession by different types of myeloid cells is necessary for the disseminated CRC cells to colonize in the liver (Fig. 8).

Recently, fibroblasts have been recognized as important constituents of the cancer microenvironment [24–26]. Further characterization revealed the heterogeneity in these cancer-associated fibroblasts (CAFs) in terms of their origins and functions [27, 28]. Interestingly, some CAFs are derived from BM cells [29, 30]. Recently, fibrocytes has been identified as progenies of monocytes [31], and are involved in the pathophysiology of various diseases associated with chronic inflammation and tissue fibrosis [22, 32]. However, cancer progression has been rarely associated with fibrocytes except a few recent reports [33, 34]. Our results show that fibrocytes are key components of the metastatic foci and can promote invasion and colonization of cancer cells through expression of MMP2, and suggest that fibrocytes might be constituents of CAFs.

We have recently shown that CCR1 plays a key role in liver metastasis of human CRC that lost SMAD4 [35]. In human CRC specimens, the level of CCL15, a chemotactic ligand for the CCR1 receptor, is inversely correlated with that of SMAD4. Moreover, higher levels of CCL15 in the primary CRC are associated with abundance of CCR1^+^ cells recruited to the metastatic foci, and with shorter disease-free survival [35]. Consistent with these observations, we have found that CD15^+^ cells specifically express CCR1 at the site of liver metastasis of human CRCs. Notably, the CD15^+^ cells at the metastatic lesions are of the mononuclear morphology despite that CD15 is expressed mostly in human neutrophils. Unlike mouse neutrophils, human circulating neutrophils do not usually express CCR1. Accordingly, it is conceivable that CCR1 is induced in myeloid cells by the microenvironment of the metastatic lesions. It is known that some cytokines can directly up-regulate CCR1 expression in human neutrophils [36, 37] although it remains to be investigated how CCR1 is up-regulated in the human liver metastatic lesions.

In summary, we have identified myeloid cells that express CCR1, MMP9 and MMP2 to promote cancer metastasis (Fig. 8). Although these findings are based on a single model with Smad4 deficient CRC, further investigation of these cancer-driven host reactions in human CRC may lead to effective preventive measures against cancer metastasis.

## Electronic supplementary material

Below is the link to the electronic supplementary material.

Supplementary material 1 (PDF 23923 kb)

## Abbreviations

*APC*: Adenomatous polyposis coli (gene)
APC: Allophycocyanin
BAC: Bacterial artificial chromosome
BM: Bone marrow
CAF: Cancer associated fibroblasts
CRC: Colorectal cancer
Φ: Diameter
ECM: Extracellular matrix
FCS: Fetal calf serum
FSC: Forward scatter
iMC: Immature myeloid cell
MMP: Matrix metalloproteinase
PDGFR: Platelet-derived growth factor receptor
PE: Phycoerythrin
PI: Propidium iodide
SSC: Side scatter
TAM: Tumor associated macrophage
TAN: Tumor associated neutrophils
