# Supplementary material for: CCR1-mediated accumulation of myeloid cells in the liver microenvironment promoting mouse colon cancer metastasis
Source: Clin Exp Metastasis. 2014 Oct 18;31(8):977–89. doi: 10.1007/s10585-014-9684-z (PMC4256518; doi:10.1007/s10585-014-9684-z)

Supplementary Fig. 1

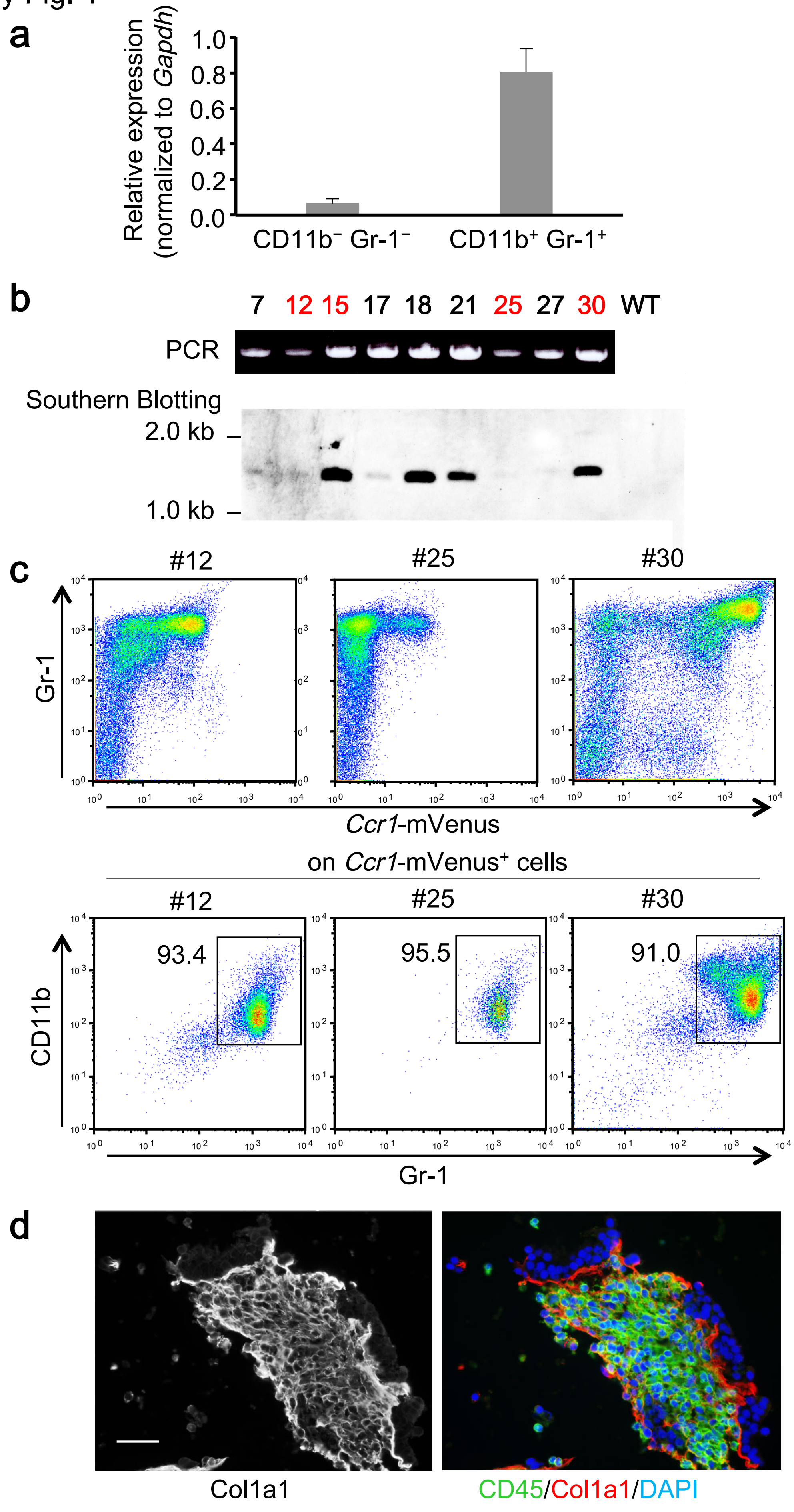

Supplementary Fig. 2

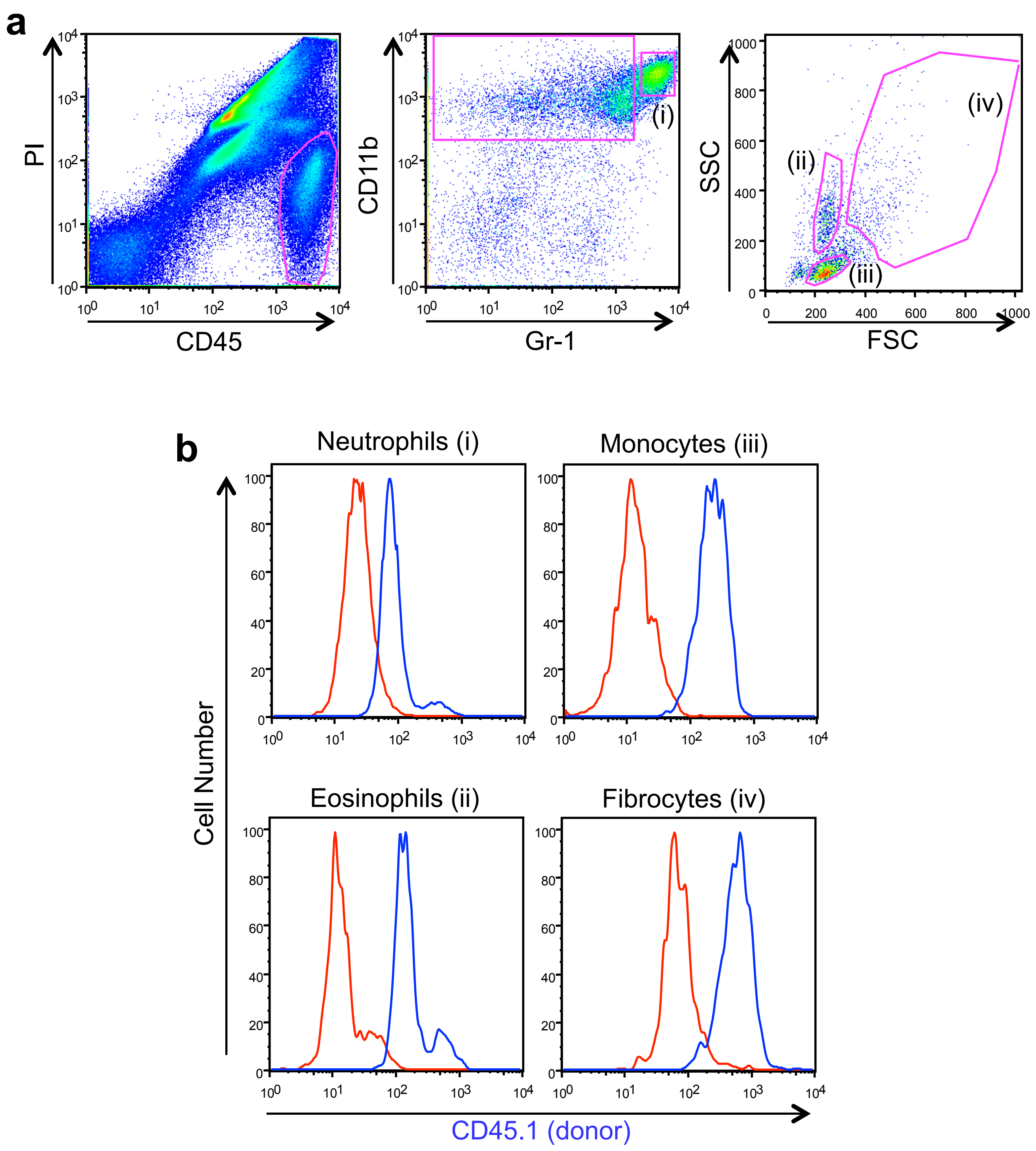

Supplementary Fig. 3

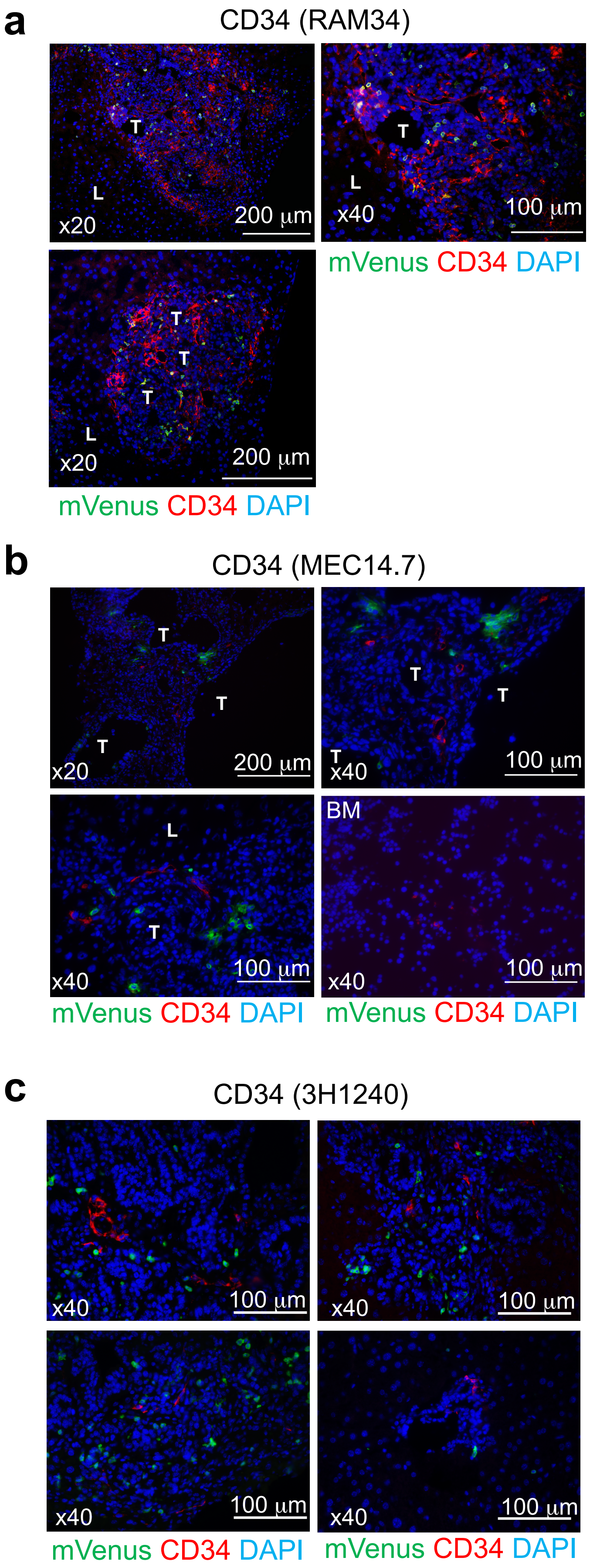

Supplementary Fig. 4

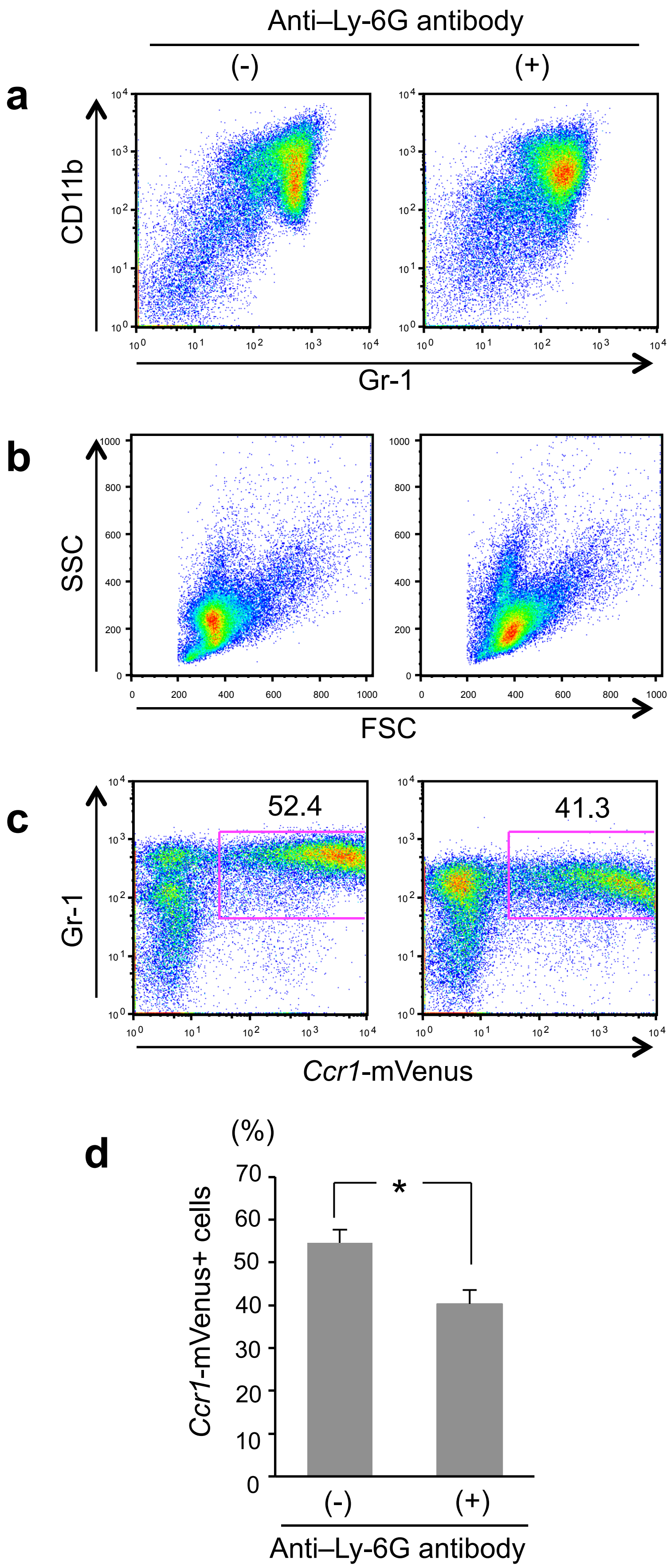

Supplementary Fig. 5

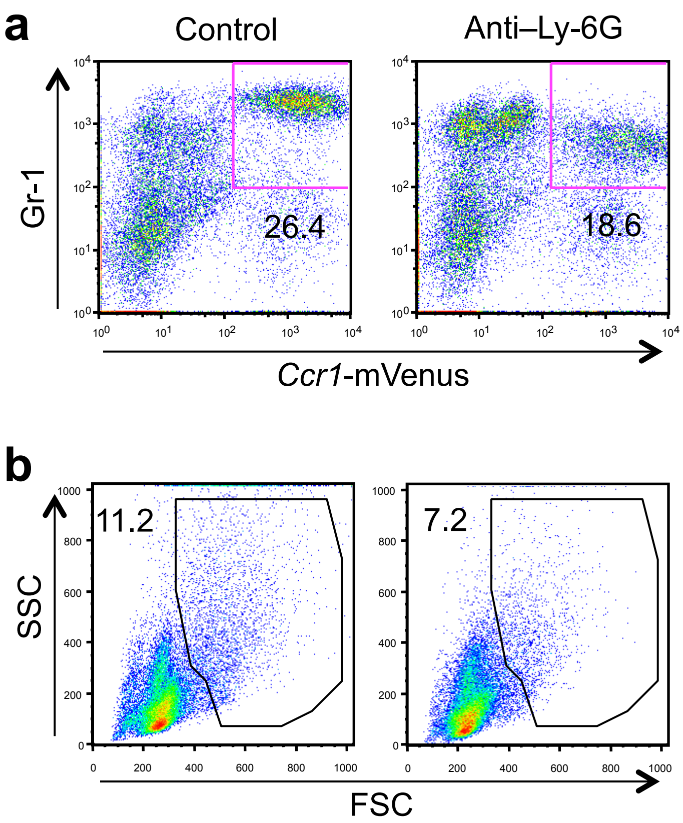

Supplementary Fig. 6

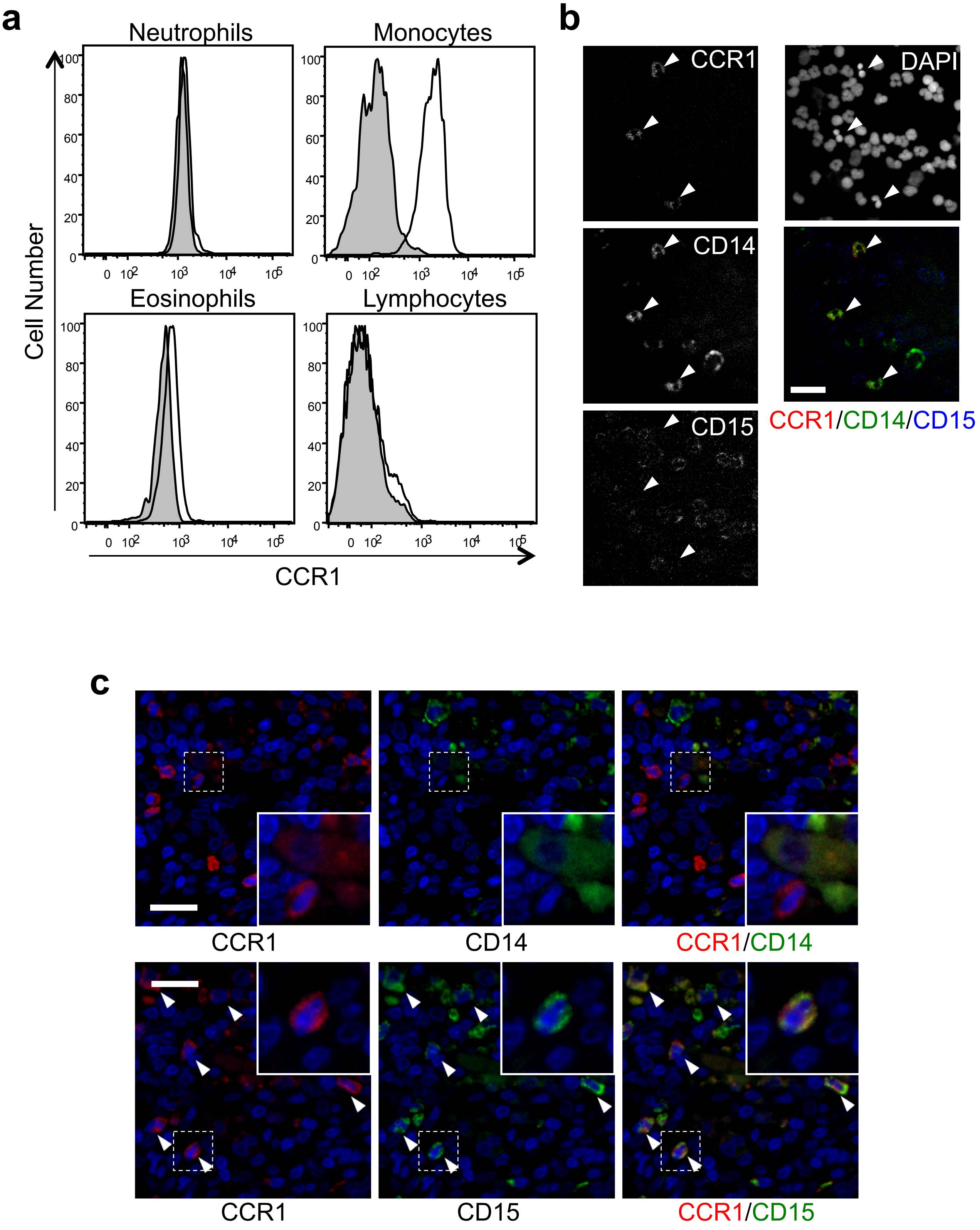

Supplement: Supplementary file 1 — Supplementary material 1 (PDF 23923 kb) [file 10585_2014_9684_MOESM1_ESM.pdf]
